# Microglial nodules provide the environment for pathogenic T cells in human encephalitis

**DOI:** 10.1007/s00401-019-01958-5

**Published:** 2019-01-20

**Authors:** Anna R. Tröscher, Isabella Wimmer, Lucía Quemada-Garrido, Ulrike Köck, Denise Gessl, Sanne G. S. Verberk, Bethany Martin, Hans Lassmann, Christian G. Bien, Jan Bauer

**Affiliations:** 1 Department of Neuroimmunology, Center for Brain Research, Medical University of Vienna, Spitalgasse 4, 1090 Vienna, Austria; 2 Epilepsy Center Bethel, Krankenhaus Mara, Bielefeld, Germany; 3 Laboratory Krone, Bad Salzuflen, Germany

**Keywords:** Rasmussen encephalitis, Microglia nodules, T-cell-mediated encephalitis, Transcriptomics, Inflammasome

## Abstract

Microglia nodule formation is a common feature in inflammatory brain diseases mediated by T lymphocytes such as viral and paraneoplastic encephalitis, multiple sclerosis, and Rasmussen encephalitis (RE). However, its role has not been fully understood yet. We hypothesized that, in RE, microglial nodules provide an environment for the initiation of the later dominating T-cell cytotoxicity. In RE stage 0, small primary microglia nodules could be identified in the absence of T cells. These primary nodules showed inflammasome activation and endosomal Toll-like receptor upregulation. In stage 1, T cells migrate into the parenchyma and intermingle with microglial cells, thereby forming secondary nodules in which neurons are destroyed. Whole-genome transcriptome analysis at this point showed upregulation of several inflammatory pathways including interferon signaling and major histocompatibility complex-I signaling. Inflammatory profiles, like the ones observed in RE, could be induced upon TLR3 stimulation in neonatal microglial cell cultures. Taken together, our results point towards activation of endosomal TLRs, resulting in increased interferon signaling, inflammasome activation, and chemokine upregulation as early steps in RE pathogenesis. This activity sets the scene for subsequent infiltration of T cells and destruction of neurons. Similar to RE, this microglial microenvironment might be a crucial step in other T-cell-mediated inflammatory brain diseases.

**Electronic supplementary material:**

The online version of this article (10.1007/s00401-019-01958-5) contains supplementary material, which is available to authorized users.

## Introduction

Microglia nodules are neuropathological characteristics of viral and autoimmune encephalitides as well as multiple sclerosis (MS) [3, 29, 30, 39, 44, 45, 47, 50]. In herpes simplex virus encephalitis and cytomegalovirus encephalitis, the microglial cells within such nodules are engaged in phagocytosis of degenerating infected neurons which are actively killed by CD8^+^ cytotoxic T lymphocytes (CTLs) [31]. In MS, microglial nodules, referred to as (pre-)active lesions, are found in periplaque normal-appearing white matter, and are associated with degenerating axons, stressed oligodendrocytes, and activated innate immunity [9, 20, 30, 45, 47, 50]. These nodules, therefore, can be regarded as local microenvironmental hotspots associated with augmented innate immunity, microglia, and T-cell communication and neurodegenerative mechanisms. Rasmussen encephalitis (RE), a unihemispheric pediatric epileptic disorder, is characterized by microglia nodule formation, CTL infiltration, and neurodegeneration [42, 44]. Patients develop focal seizures and brain atrophy, leading to hemiparesis, hemiplegia, or hemianopia [6, 44, 51]. RE is one of the few exclusively CTL-mediated diseases, where neurons are specifically targeted [5]. Moreover, clonal expansion of restricted T-cell population in the brain of RE patients was shown [46]. Although the etiology of RE is still unknown, the CTL apposition around neurons and clonal T-cell expansion points towards the recognition of a specific epitope. Besides the adaptive immune response, the role of the innate immune system in RE is not well understood yet. A few studies have analyzed the expression of inflammatory mediators, such as chemokines and cytokines. Several T-cell-attracting chemokines were shown to be upregulated, namely CCL5, CXCL9, and CXCL10 [41]. Moreover, inflammasome-related genes, such as interleukin (IL)-1β and interferon (IFN)-γ, were shown to be upregulated in RE [28, 36, 43]. Unfortunately, all these studies were performed on highly inflamed disease stages and do not answer the question about the underlying disease mechanisms at the beginning of the disease. Furthermore, it is unclear how T cells gain access to the CNS, at which point microglia are activated and how these cells interact.

In this study, we addressed these questions by taking advantage of the fact, that one RE patient can show various pathological differentiated lesions. We classified resected brain tissue according to the cortical pathology staging from Pardo et al. and could, therefore, select lesions ranging from very early to acute stages [42]. In the earliest stage (stage 0), neither neurodegeneration nor CTL infiltrates are present. In the intermediate stage (stage 1), local T-cell infiltrates together with microglia nodules can be found. In these areas, focal neurodegeneration has already occurred. In the acute stage (stage 2), T-cell infiltrates are extensive, with big multifocal infiltrates and high numbers of T cells throughout the tissue. Microglia activation as well as neurodegeneration are severe and panlaminar [42]. We further performed whole-genome transcriptome analysis of the different stages and age-matched control samples. Gene set enrichment analysis revealed an early upregulation of innate immune responses, such as major histocompatibility complex (MHC)-class I-mediated antigen presentation and increased IFN signaling. Only later pathways linked to T-cell signaling were increased. In parallel, we could show that the very first histopathological changes in RE are small microglia nodules, consisting of only 3–7 cells, which we termed primary nodules (PN). They already show activated innate immunity, by an upregulation of inflammasome genes and endosomal Toll-like receptor (TLR) 7. Only later T cells infiltrate into the brain parenchyma and migrate to the primary nodules, where they intermingle with the activated microglia and form secondary nodules (SN). The T cells change the inflammatory milieu within the nodule and secrete IFN, which in turn leads to IL-1β secretion, known for its pro-epileptogenic properties [52]. To verify endosomal TLRs as possible initiator of the inflammatory reactions in RE, we compared gene expression of in vitro Poly(I:C)-stimulated neonatal microglia to RE. The early stages of RE resembled poly(I:C)-stimulated microglia, especially with respect to T-cell-attracting chemokines and anti-viral immune response.

These findings point towards a two-step inflammatory process: first, microglia are activated by TLR signaling and upregulate inflammasome genes, T cell- and monocyte-attracting chemokines. After the infiltration, CD8^+^ T cells attack neurons and modulate the inflammatory milieu by IFN-γ secretion, thereby promoting inflammation. This may lead to the expansion from focal inflammatory hotspots to panlaminar inflammation and neurodegeneration at later stages. Moreover, these findings are not only important for RE, but allow a possible insight into other CTL-mediated diseases, such as paraneoplastic encephalitis or MS, where the early stage tissue is not available.

## Materials and methods

### Patients

This study was performed on 4% neutral buffered formalin-fixed and paraffin-embedded (FFPE) resected brain tissue which was collected at the epilepsy center Bethel, Bielefeld, Germany, between 1991 and 2015. A total of 30 FFPE tissue blocks from 27 different patients were used. Of these, 23 patients were diagnosed with RE and underwent epilepsy surgery. As control material age-, gender-, and area-matched (temporal cortex) controls were used from patients suffering from low-stage tumors, such as dysembryoplastic neuroepithelial tumors (DNT), gangliogliomas, or cavernomas. Tissue blocks from these patients were tumor-free as analyzed by a trained epilepsy neuropathologist. A summary of patients’ demographic data is given in Table 1. Detailed information on single patients is shown in Supplementary Table 1.

**Table 1:** Summary of patients’ demographic data: gender distribution was tested with the Fisher’s exact test and differences in age and disease duration with a two-tailed *t* test

Characteristic	RE	Controls	*p* value
Gender (m/f)	(5/8)	(3/4)	1.00
Mean age (years)	7.9	7.4	0.68
Disease duration	2.7	4.8	0.15

### Ethics statement

Patients gave informed consent to research use of their brain tissue. The study was approved by the ethical committees of the medical University of Münster (Number 2015-088-f-S) and the Medical University of Vienna (Number 1206/2013).

### Histopathological evaluation and staging

Low malignant tumor FFPE blocks without histopathological changes (no tumor infiltration) were further analyzed for immunopathological evaluation of usability. Immunolabelling for CD68 was performed to investigate any inflammatory reaction indicated by microglia activation. Moreover, NeuN was used to exclude cases with the signs of neurodegeneration. As we were also interested in epileptogenic changes, we investigated the tissue for seizure activity by staining for c-Fos as marker for neuronal activity. Only tissue blocks with the absence of inflammation, neurodegeneration, or seizure activity were included in the control group.

All RE cases were investigated for neurodegeneration, microglia activation, and CD3^+^ T-cell infiltrates, and were staged according to Pardo et al. [42]. As the main aim of this study was to investigate the early changes in the pathogenesis of the disease, FFPE blocks ranging in stage 0 (normal-appearing cortex), 1 (early stage), or 2 (intermediate stage) were selected.

### RNA isolation of FFPE material

Three 7 µm sections were cut; the middle section was stained for H&E and used as a template to delineate cortical grey matter, while the other two were used for RNA isolation. Tissue dissection was performed manually using the H&E template. Collected tissue was deparaffinized according to the standard protocols followed by RNA isolation using the High Pure FFPE RNA Micro Kit (Cat. No. 04823125001; Roche), according to the previously established protocol [54].

### RNA quality control and quantification

RNA quantity and integrity of each sample was determined with the Agilent 2100 Bioanalyzer using the Agilent RNA Pico Chips. The DV200, a quality parameter for RNA derived from FFPE [54], was calculated using the 2100 Expert software (Supplementary Table 1) (version B.02.08.SI648 (SR2)).

For further RNA quality determination, real-time PCR (qPCR) was performed with two standard house-keeping genes, glyceraldehyde 3-phosphate dehydrogenase (*GAPDH*) and succinate dehydrogenase complex flavoprotein subunit A (*SDHA*). For this, 8 ng total RNA were transcribed into cDNA using iScript™ cDNA Synthesis Kit (Cat. No. 1708890; Bio-Rad) according to the manufacturer’s instructions. For qPCR, cDNA template (corresponding to 200 pg RNA) was mixed with SsoAdvanced™ Universal SYBR^®^ Green Supermix (Cat. No. 1725270; Bio-Rad), forward and reverse primers (each in a final concentration of 200 nM), and nuclease free water to a final reaction volume of 10 µl. qPCR was then performed on a Step One Plus™ Real-Time PCR System (Applied Biosystems™, Thermo Fisher Scientific) using the following thermal cycling conditions: 30 s at 95 °C, 40 cycles alternating 15 s at 95 °C and 30 s at 60 °C. Afterwards, routine melting curve analysis was performed. Threshold settings were adjusted to 0.3 for both house-keeping genes (Applied Biosystems StepOne Software v2.3). Successful amplification of both genes of each sample was considered as good RNA quality for further downstream processes.

### Affymetrix GeneChip™ whole-genome microarrays

2 ng total RNA input was used for sample preparation as previously described [54]. Subsequently, samples were hybridized to GeneChip™ Human Gene 2.1 ST 24-Array Plate (Affymetrix, Thermo Fisher Scientific, Cat. Nr.: 902136), which were then scanned with GeneTitanTM MC Instrument (Affymetrix, Thermo Fisher Scientific). The above-mentioned steps were performed at the Genomic Core Facility of the Medical University of Vienna. Resulting CEL files were loaded into Affymetrix Expression ConsoleTM software (v1.4.1.46) and normalized by the RMA-Sketch algorithm. Analysis of differentially expressed genes was performed with the Affymetrix Transcriptome Analysis Console (TAC 4.0.0.25). For gene set enrichment analysis (GSEA), the data set of differentially expressed genes (± 1.5-fold change with a *p* value below 0.05) was submitted to the reactome pathway analysis tool (version 64) [14, 15]. In addition, functional annotation clustering (FAC) was performed with the online tool DAVID [21, 22], to verify the results from the GSEA (version 6.8).

**Fig. 1:**
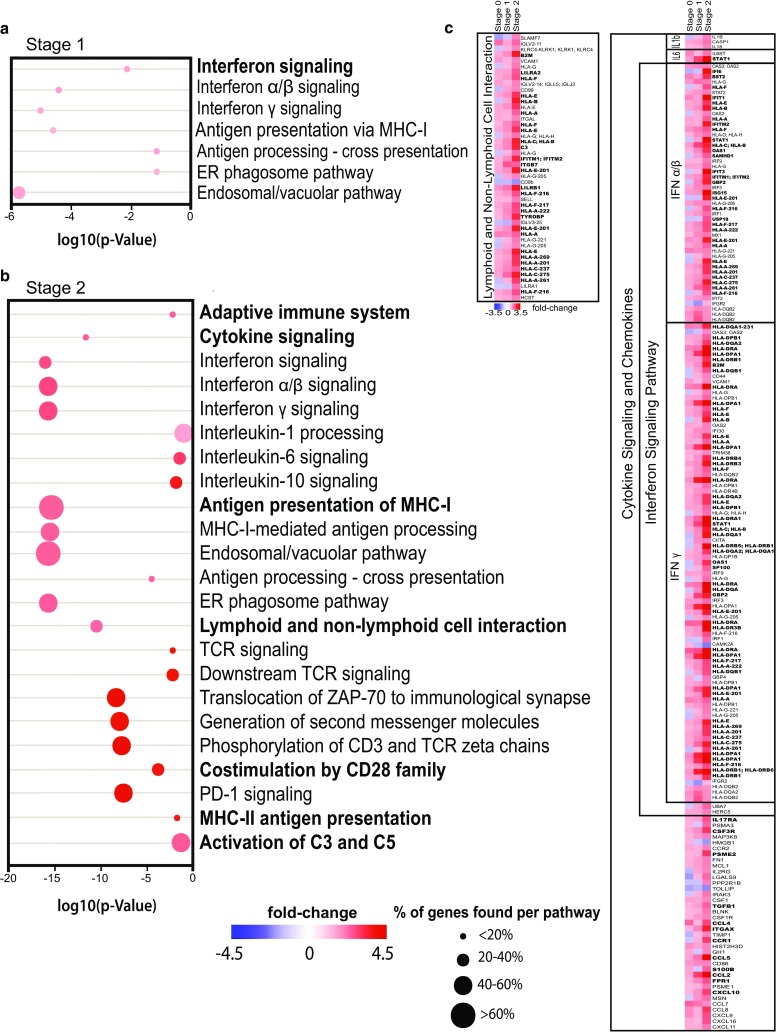
Gene set enrichment analysis (GSEA) of RE stage 1 and stage 2 reveals an upregulation of inflammatory pathways. **a** GSEA of differentially expressed genes in RE stage 1 compared to controls. **b** GSEA of differentially expressed genes in RE stage 2 compared to controls. The color of each dot represents linear fold change; the size of the dot indicates percentage of genes represented in this data set compared to all genes associated with this pathway. The location of each dot represents the log10(p value) of the GSEA of each pathway, only significantly differentially expressed pathways are depicted. Main pathways are indicated in bold; subsidiary pathways are indicated regularly. **c** Detailed representation of selected pathways and the differentially expressed genes (transcript cluster IDs shown) represented in our dataset. Fold changes were calculated in comparison to controls and are represented in color code. Genes of each pathway, which are among the leading edge of overall differentially expressed genes (top 500) are indicated in bold. Controls (*n* = 7), stage 0 (*n* = 5), stage 1 (*n* = 6), and stage 2 (*n* = 6)

Microarray data, which were used for the generation of Fig. 1 and parts of Fig. 6a, were deposited in NCBI’s Gene Expression Omnibus GSE121010.

### Validation of differentially expressed genes by qPCR

qPCR was used to validate the gene expression of *IL1B*, *IL18*, *CASP1*, *TLR3,* and *TLR7* in controls and RE samples. The house-keeping gene *GAPDH* was used as reference gene. To exclude normalization bias introduced by a different degradation speed of mRNA between 5′ and 3′ end, the amplicon of the target gene and house-keeping gene were always from the same end of mRNA (either 5′ or 3′). Therefore, two different GAPDH primers were used (primer specifications in Supplementary Table 8). qPCR was performed according to the protocol described above, with the exception of 50 cycles run for inflammasome genes and 60 cycles for TLRs. In many control cases, no amplification product was obtained for the gene of interest as the expression rate was below the detection threshold. We, therefore, manually adjusted the Ct value to the maximum number of cycles +1 cycle. As fold change cannot be calculated without amplification of the target gene in the control group without introducing tremendous bias, only ΔCt was calculated for normalization [17]. As non-detects were adjusted manually and do not reflect the true expression value, non-parametric statistics was performed to account for this fact [17]. As ΔCt values are indirect proportional to the actual expression values (the higher the ΔCt, the lower the expression value), resulting values were further multiplied with − 1 for a better graphical representation and more intuitive data interpretation.

### Immunohistochemistry

Immunohistochemical stainings were performed as described elsewhere [4]. T cells (CD3), microglia (CD68), and neurons (NeuN) were stained for the staging of RE cases. For the selection of the control samples, c-Fos, as a marker for neuronal activation, was additionally stained to exclude epileptic activity of the brain area. Moreover, stainings were performed for IL-1β, IL-18, caspase-1, TLR7, and pSTAT1. For the differentiation of microglia and peripheral macrophages, TMEM119 staining was performed [10]. To evaluate the homeostatic state of the microglia, we used P2RY12, kindly provided by Dr. Butovsky [10]. To evaluate the activation status of microglia, an antibody against HLA-DR was used. Antigen retrieval was performed by heating the sections for 1 h in EDTA (0.05 M) in tris(hydroxymethyl)aminomethane (Tris) buffer (0.01 M, pH 8.5) or citrate buffer (0.01 M, pH 6) in a household food steamer device (details regarding staining procedures see Supplementary Table 9).

### Fluorescence staining

Fluorescent double and triple labelling was performed for TLR7 and cell markers as well as for pSTAT1, CD3 and Iba1 as well as for T-bet, CD3, and Iba1. To this end, slides were treated according to the protocol used for multiple antibodies from same species as described previously [4]. In short, pSTAT1 or T-bet antibody was applied overnight, followed by a corresponding biotinylated secondary system and tyramide enhancement. Slides were then again steamed in EDTA (0.05 M) in Tris buffer (0.01, pH 8.5) for 30 min, followed by 1 h incubation with Cy2-conjugated streptavidin. In a second overnight incubation, CD3 and Iba1 primary antibodies were applied to the tissue together, followed by Cy3- and Cy5-conjugated secondary antibodies.

### Quantification of cells

Quantification of cells expressing the protein of interest (IL-1β, IL-18, caspase-1, and TLR7) in the cortical grey matter (1–2.5 mm^2^) was performed with an ocular grid in 400 × magnification and the number of cells per mm^2^ was calculated. Quantification of PN was performed in cortical grey matter of stage 0 cases and controls with an ocular grid in 100 × magnification and PN per mm^2^ were calculated. Cell quantification for correlations of different cell types and proteins expressed within a nodule were performed by counting cells of interest in a 400 × magnification in each nodule separately.

### Densitometry

To specifically quantify the differences in protein abundance of caspase-1, IL-18 and TLR7, we determined staining intensity of microglia of controls and stage 0 samples. To this end, we used the Image ProPremier software (version 9.3) and used the automated cell counting function with a manually set threshold. We also performed background correction to eliminate bias from unspecific staining of areas without positive cells.

### Statistics

For statistical analysis, GraphPad Prism 6 was used. As our human data are unevenly distributed, non-parametric tests were used. For analysis of qPCR and cell quantification data, Kruskal–Wallis test with multiple comparisons and Dunn’s post hoc correction were used. Correlations between T cells and microglia, pSTAT1^+^ and IL1β^+^ cells, and t-bet and microglia were calculated using Spearman correlation test. Densitometric measurements were analyzed using a two-tailed Mann–Whitney test. All graphical data are presented as median and interquartile range. *p* values smaller than 0.05 were considered as significant. Heat maps of differentially expressed genes and hierarchical sample clustering were created using the MeV software (MeV4.8.1., TM4).

### Primary microglial cell culture

#### Animals

Lewis rats were bred at the Decentral Facilities of the Institute for Biomedical Research (Medical University of Vienna).

### Microglia cultures

Microglia cultures were performed as previously described [16, 19, 25]. In short, new-born Lewis pups were decapitated and the brains dissected and homogenized in growth medium (RPMI/10% fetal calf serum/1% l-glutamine/1% Pen/Strep). Mixed glia cultures were incubated for 10 days on poly-l-lysine-coated flasks (37 °C, 5% CO_2_). Microglia and progenitor cells were detached from the astrocyte base layer by shaking the culture flasks for 10–12 h at 125 rpm at 37 °C. Supernatants were collected and 2.5 × 10^5^ cells/well were plated in 24-well plates. Flasks were incubated for 30–40 min to allow for microglia to attach to the plastic. Subsequently, contaminating cells were removed by washing with PBS. Microglia cells were incubated in growth medium for 24 h prior to stimulation experiments. From the mixed glial cultures, a total of three shake-offs were performed (every 3–5 days). For this study, only the second and the third shake-off were used.

### Microglia stimulation

For the activation of TLR3- and TLR4-signaling pathways, cells were incubated for 12 h with 10 µg/ml Poly(I:C) (Cat. Nr.: 4287, Tocris) and 10 ng/ml LPS from *Salmonella enterica typhimurium* (L6143-1MG, Sigma-Aldrich), respectively.

### RNA isolation

RNA was isolated using the RNeasy Micro Kit from Qiagen (Cat. Nr.: 74004) according to the manufacturer’s instructions. RNA quality and quantity was determined using the Agilent 2100 Bioanalyzer RNA Nano Chips (Cat. Nr.: 5067–1512).

### Gene expression analysis of microglial cells

For a broad overview of inflammatory gene expression by stimulated microglia, the “Cytokines and Chemokines” RT^2^ Profiler PCR Arrays (Qiagen, Cat. Nr.: 330231) were performed according to the manufacturer’s instructions. 75 ng total RNA were transcribed using the RT^2^ First Strand Kit (Qiagen, Cat. Nr.: 330401). For the PCR arrays, the RT^2^ SYBR Green ROX qPCR Mastermix (Cat. Nr.: 330522) was used and PCR cycles were programmed according to the manual (10 min polymerase activation at 95 °C, followed by 40 cycles of 15 s 95 °C and 1 min at 60 °C), followed by routine melting curve analysis.

### Data analysis and statistics

To enable the comparison of gene expression between human microarray and rat qPCR data, z scores were calculated for all genes of interest for which a baseline expression level was detected in the rat qPCR arrays. Significant differences upon microglia stimulation were routinely analyzed by one-way ANOVA. Heat maps of differentially expressed genes were created using the MeV software (MeV4.8.1., TM4).

## Results

### Whole-genome transcriptomic analysis reveals initial anti-viral immune response

As a first step, whole-genome transcriptome analysis provided a comprehensive overview on differentially expressed genes in RE (Fig. 1, Supplementary Table 2). In stage 0 a total of 630 differentially expressed genes were detected (296 upregulated, 334 downregulated with a minimum of ± 1.5 fold change, and *p* value ≤ 0.05); however, gene set enrichment analysis (GSEA) did not reveal differentially expressed pathways compared to controls (Supplementary Table 2). In later histopathological stages, inflammatory signaling pathways became increasingly upregulated. In stage 1 cortex, pathological changes are limited to focal areas with mild inflammation and degeneration of single or few neurons [5, 42]. In these stage 1 cortici, pathways associated with MHC-class I-related antigen processing/presentation, such as the endosomal/vacuolar pathway and antigen processing-cross presentation, and IFN signaling were significantly upregulated (Fig. 1a). In stage 2, inflammation is much more pronounced and neuronal degeneration is multifocal or even panlaminar [42]. At this stage, GSEA revealed differential expression of inflammatory pathways including cytokine signaling, T-cell signaling, MHC-class II antigen processing, and activation of complement components (Fig. 1b). Since we were interested in the early pathological events, we focused on pathways linked to spreading of inflammation and recruitment of peripheral immune cells, namely cytokine and chemokine signaling as well as lymphoid and non-lymphoid cell interactions. From those, in stage 1, IFN-α/β and IFN-γ signaling (+ 1.5-fold) showed the highest percentage of entities represented in our data set (Fig. 1a, c). Also in stage 2, IFN signaling was highly prominent (IFN-α/β signaling + 2.5-fold; IFN-γ signaling + 2.7-fold). IL-1 processing was increased 1.7-fold (Fig. 1b, c). The lymphoid and non-lymphoid cell interaction cluster, important for interactions of CTLs with microglia and neurons, showed a strong (2.3-fold) increase in stage 2 (Fig. 1b, c). Analysis details of GSEA are indicated in supplementary Table 2. Additionally performed FACs results from stage 1 and 2 are indicated in supplementary Tables 3–7, which found similar enriched pathways as the GSEA.

### Microglial activation precedes T-cell influx and is characterized by inflammasome activation

**Fig. 2:**
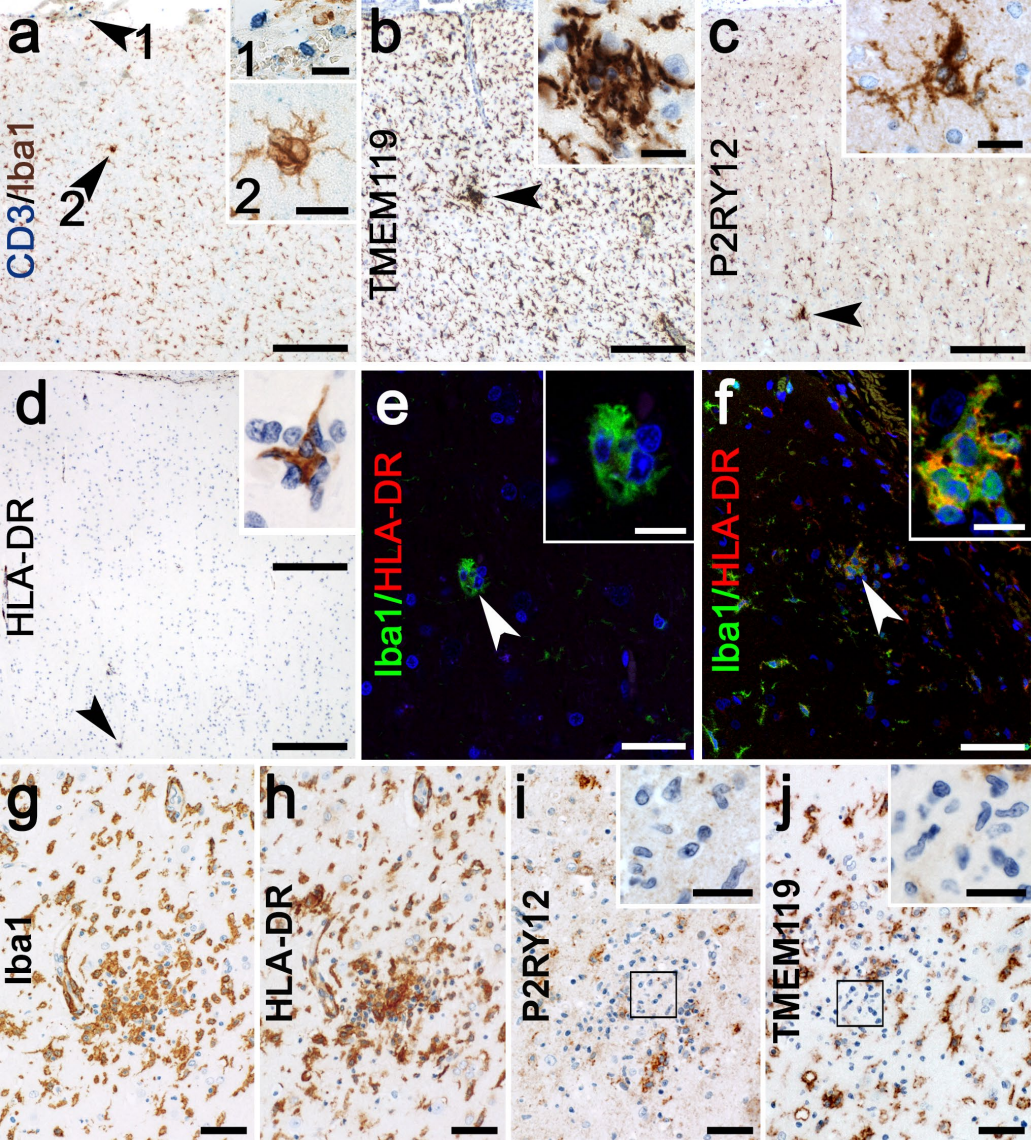
Microglial activation in different stages of RE. **a** Microglia form small nodules in the absence of T cells in the parenchyma, only a few T cells are found in the meninges (inset 1) and a PN in the cortical grey matter (inset 2). PNs are strongly positive for homeostatic marker, **b** TMEM119 and **c** P2RY12. **d** PNs positive for microglial activation marker HLA-DR are found in areas devoid of HLA-DR upregulation around the PN. **e** small PNs can be HLA-DR negative, but **f** most express HLA-DR. **g** large SN in stage 2 shows, **h** drastic upregulation of HLA-DR, but a downregulation of homeostatic markers **i**, P2RY12, with microglia in the core of the nodule which do not express P2RY12 (inset), and **j** TMEM119, again with negative microglia in the core of the nodule (inset). Scale bars in **a**–**d** correspond to 100 µm in the main picture and 20 µm in the insets. Scale bars in **e** and **f** correspond to 25 µm in the main picture and 10 µm in the insets. Scale bars in **g**–**j** correspond to 50 µm and 20 µm in the insets

**Fig. 3:**
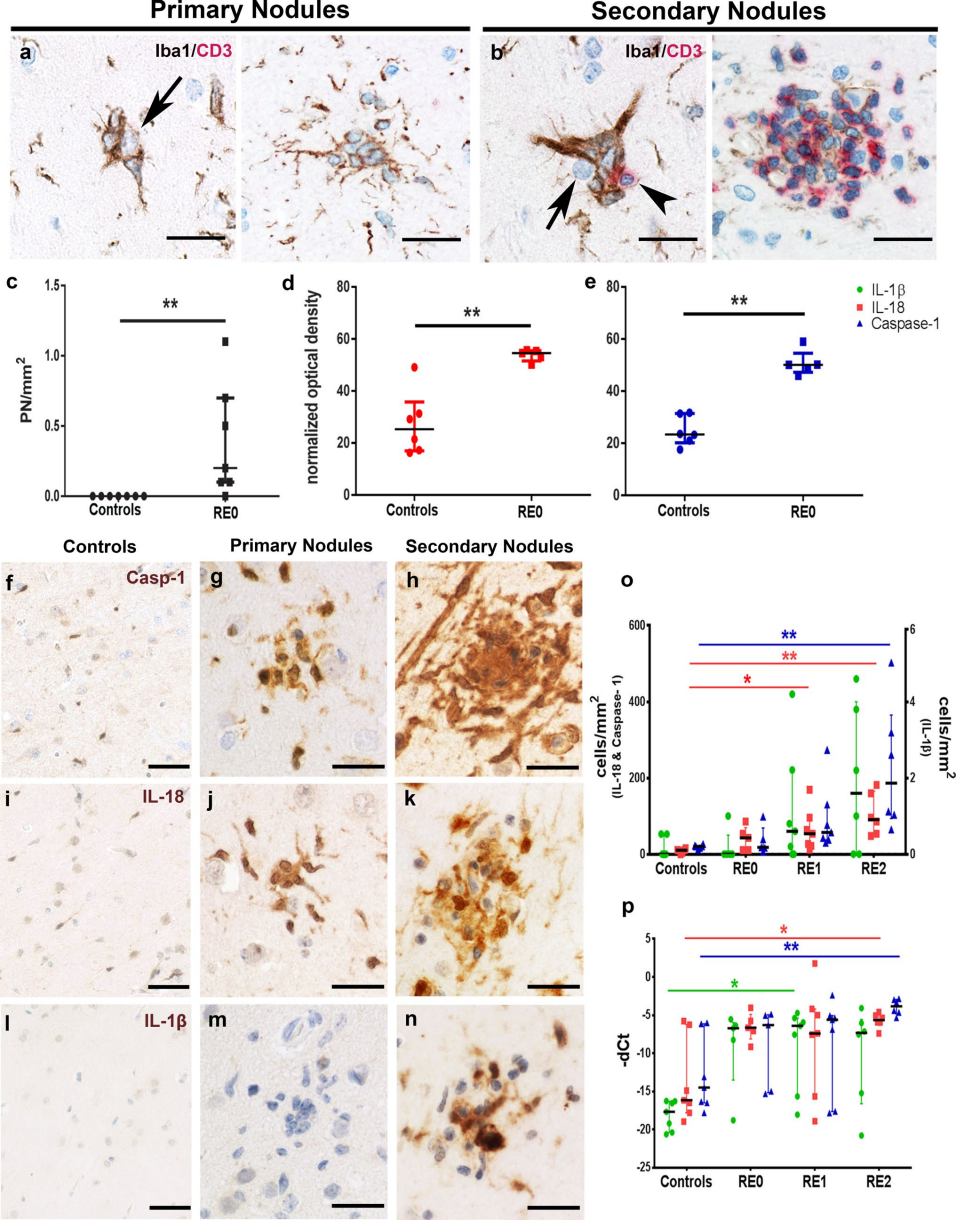
Primary nodules are the first histopathological manifestation in RE and precede T-cell infiltration. **a** PNs are small microglia clusters devoid of T cells, which form around neurons (arrow). **b** In later histopathological stages, T cells infiltrate microglia nodules (arrowhead), often around neurons (arrow). **c** Quantification of PNs in controls (*n* = 7) and stage 0 cases (*n* = 5). **d** Densitometric measurements for IL-18 in microglia of controls (*n* = 7) and microglia-forming PNs in stage 0 (*n* = 5). **e** Densitometric measurements for caspase-1 in microglia of controls (*n* = 7) and microglia-forming PNs in stage 0 (*n* = 5). (Two-tailed Mann–Whitney test), representative pictures of caspase-1, weakly expressed in **f** controls, increased in **g** PN and further in **h** SN. IL-18 is weakly present in **i** controls, and increased abundancy in **j** PN and **k** SN. IL-1β is not found in **l** controls and **m** PN, but is highly abundant in **n** SN. The inflammasome activation is indicated by **o** cell quantification and **p** qPCR [controls (*n* = 7), stage 0 (*n* = 5), stage 1 (*n* = 7), stage 2 (*n* = 6)]. (Kruskal–Wallis Test with Dunn’s post hoc correction for multiple testing, *p* values indicated originate from the multiple comparisons, *p < 0.05, **p < 0.01). Data are represented as median with interquartile range. Scale bars in **a**, **b** represent 20 µm, in **c**, **f**, **i** 50 µm and in **d**, **e**, **g**, **h**, **j**, **k** 25 µm

In parallel to microarray analysis, we investigated histopathological changes of microglia and T cells in the early stages of RE using the identical cortical areas. In stage 0 samples, in which infiltrating CTLs and neuronal degeneration are absent (Fig. 2a), we observed a few small nodules consisting of 3–7 microglia (Figs. 2, 3a) which were absent in the control cases (Fig. 3c). We termed these primary nodules (PN). Although these microglia were positive for homeostatic markers TMEM119 (Fig. 2b) and P2RY12 (Fig. 2c), they were also positive for activation marker HLA-DR (Fig. 2d, f) in areas where surrounding microglia were still HLA-DR negative. However, some small PNs were HLA-DR negative (Fig. 2e).

In stages 1 and 2, these primary nodules are found together with larger secondary nodules (SN) consisting of both microglial cells and T cells (Fig. 3b). In these SN, neurons are eliminated by CTLs and phagocytosed by the surrounding microglia [5]. These SNs and surrounding microglia showed strong expression for Iba1 (Fig. 2g) and HLA-DR (Fig. 2h). The specific microglial markers P2RY12 and TMEM119 were still present in many of the SN. In some of the largest SNs, part of the microglial cells, however, appeared negative (Fig. 2i, j). Based on the GSEA data, we stained PNs and SNs for inflammasome-associated molecules such as caspase-1, the enzyme cleaving pro-IL-1β and pro-IL-18. In control brains, only a few faintly labeled caspase-1^+^ microglia were found (Fig. 3f, o). In stage 0 of RE, caspase-1 intensity became stronger, which was clearly present in PNs and some of the other microglial cells (Fig. 3e, g). By densitometric measurements, we could show that caspase-1 is significantly more abundant in microglia-forming primary nodules compared to microglia from controls (Fig. 3e). Caspase-1 became even more abundant in microglia and in the SNs of stages 1 and 2 (Fig. 3h). Quantification of caspase-1^+^ cells revealed a continuous increase from stage 0–2, at which point it was significantly elevated compared to controls (Fig. 3o). IL-18, too, was weakly present in controls (Fig. 3i, o), but showed strong expression in PNs (Fig. 3j), which was also quantified by densitometric measurements of microglia-forming PN and microglia of control samples (Fig. 3d). IL18 abundancy became even more pronounced in SNs (Fig. 3k). Quantitatively, numbers of IL-18^+^ cells were significantly elevated in stages 1 and 2 (Fig. 3o). Interestingly, we could find IL-1β immunoreactivity neither in controls (Fig. 3l) nor in stage 0 PNs (Fig. 3m). In stage 1 and 2 of RE, however, IL-1β^+^ microglia were present in SNs (Fig. 3n). Although increased from stages 0 to 2 (Fig. 3o), the numbers of IL-1β^+^ cells were about 100-fold lower than caspase-1^+^ and IL-18^+^ cells. qPCR for pro-IL-1β, pro-IL-18, and caspase-1 verified the increased gene expression during stage 1 and 2 and, additionally, showed an early transcriptional activation as early as stage 0. Compared to controls, IL-1β showed a significant upregulation in stage 1. IL-18 and caspase-1 were, in contrast to IL-1β, constitutively expressed and, therefore, also present in controls, leading to a significant increase only in stage 2 (Fig. 3p).

### T-cell influx orchestrates secondary inflammatory response

**Fig. 4:**
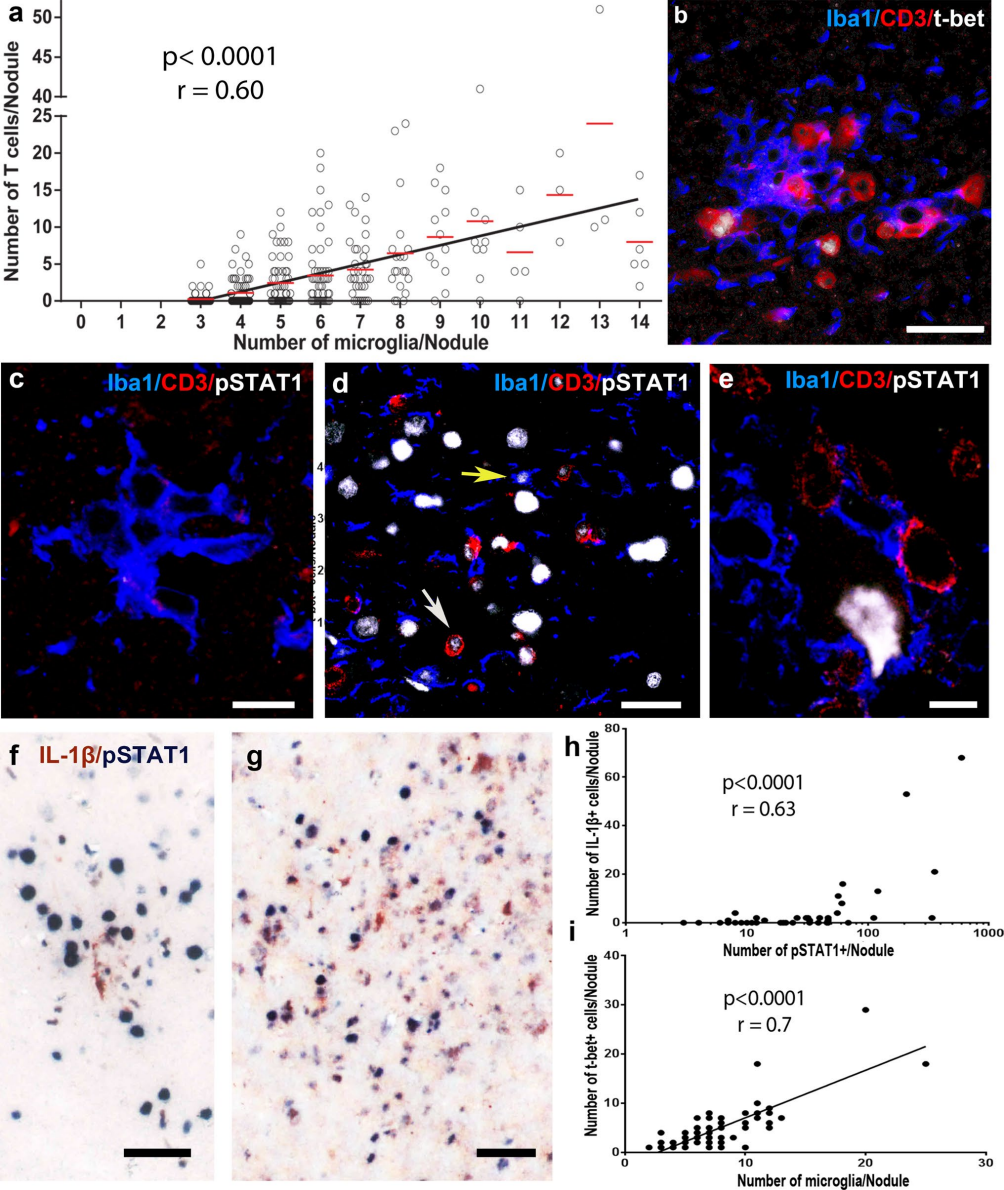
Secondary nodules are characterized by IFN-γ-producing CTLs. **a** The number of CTLs and microglia within a nodule is direct proportional (*n* (FFPE *n*(nodules) = 336 from 20 patients) Spearman correlation, *r* = 0.60, *p* < 0.0001). **b** Triple labeling of microglia (Iba1), T cells (CD3) and T-bet as a marker for IFN-γ-producing cells. Triple labeling of microglia (Iba1), T cells (CD3), and pSTAT1, the phosphorylated transcription factor for IFN signaling in **c** PN, **d** SN, where pSTAT1 is visible in T cells (white arrow) and microglia (yellow arrow) and **e** a neuron with T-cell apposition. Double labeling of pSTAT1 and IL-1β in **f** a small SN with only one IL-1β^+^ cell and **g** a big SN with many IL-1β^+^ cells. **h** The number of IL-1β^+^ cells is directly proportional to pSTAT1^+^ cells [n(nodules) = 48 from 8 patients], Spearman correlation, *r* = 0.63, *p* < 0.0001). **i** The number of t-bet^+^ cells correlates positively with the number of microglia within a nodule, (*r* = 0.7, *p* < 0.0001, *n* (single nodules) = 67 from 4 patients). Scale bars in **b** and **d** correspond to 25 µm, in **c** and to 10 µm, and in **f** and **g** to 50 µm

As mentioned above, the qualitative difference between small PNs and large SNs is the presence of T cells. To illustrate this, we quantified the composition of microglial nodules by counting the number of microglial cells and CTLs in individual nodules. This revealed a positive correlation between the numbers of microglia and CTLs within nodules (Fig. 4a). Moreover, the presence of CTLs drastically changed the inflammatory profile of the nodular microenvironments. T-bet, a marker for IFN-γ producing CTLs, and phosphorylated STAT1 (pSTAT1), the activated downstream transcription factor of IFN signaling, were absent in PNs (Fig. 4c), but were strongly expressed in SNs (Fig. 4b, d, e). Moreover, the number of t-bet^+^ cells was directly proportional to the number of microglia within a nodule (Fig. 4i). Within SN, pSTAT-1 expression was detected in various cells including T cells, microglia, and neurons (Fig. 4d, e). Interestingly, we observed that IL-1β^+^ cells were only present in nodules positive for pSTAT1 (Fig. 4f, g). Quantitatively, the number of pSTAT1^+^ cells positively correlated with the number of IL-1β^+^ cells (Fig. 4h).

### Toll-like receptor signaling initiates inflammatory response

**Fig. 5:**
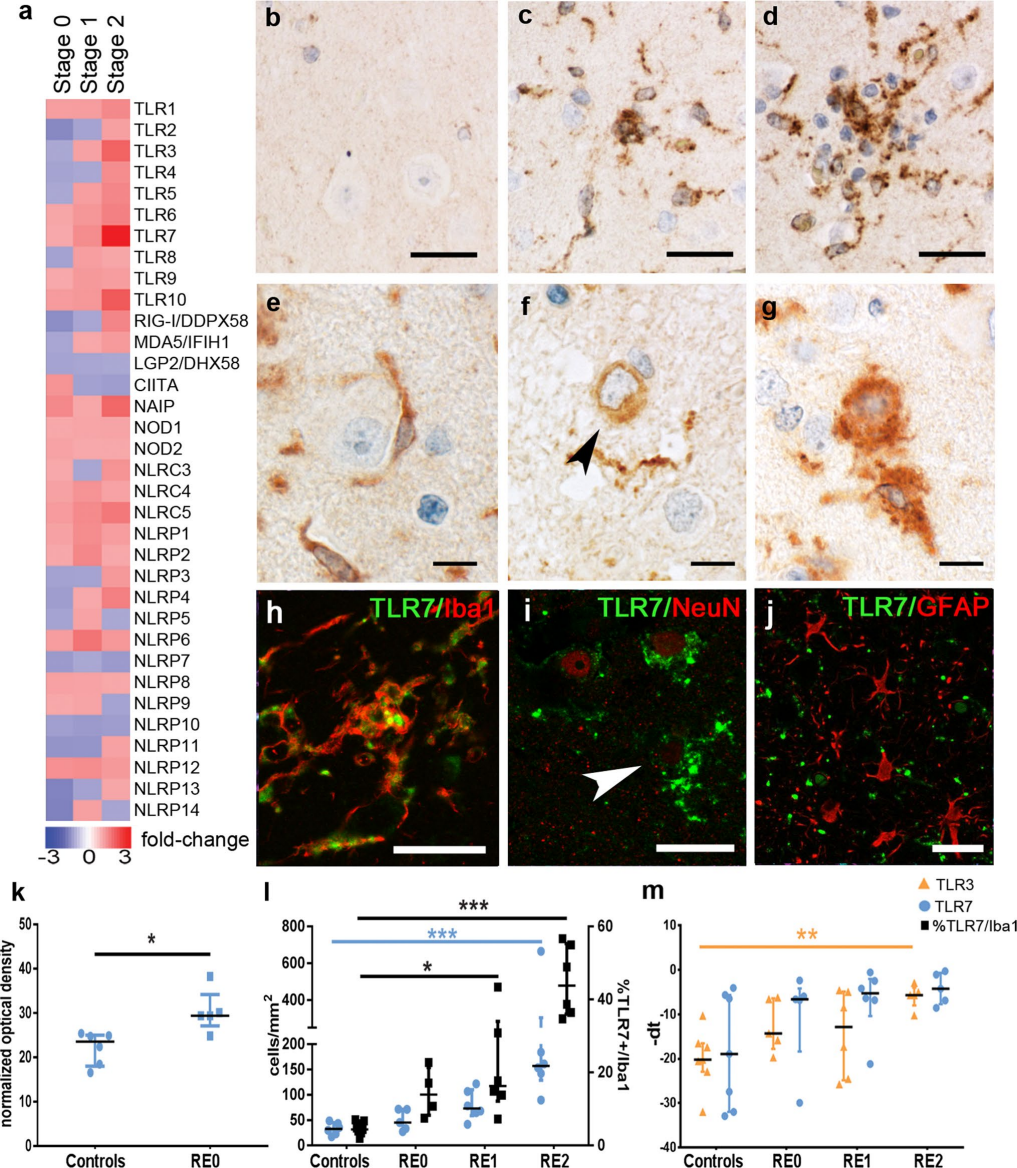
PAMPs and DAMPs involved in the pathogenesis of RE. **a** PAMPs and DAMPs expression values indicated in linear fold changes compared to controls. **b** Controls only show very faint TLR7 immunoreactivity, whereas **c** PN show increased expression of TLR7, which is also present in **d** SN. **e** A healthy neuron without TLR7 reactivity, in contrast to perineuronal microglia attached to it. **f** A neuron with TLR7 reactivity but healthy nucleus and **g** an apoptotic neuron with intense TLR7 reactivity. **h** Microglia (Iba1) and TLR7 fluorescent stainings overlap, indicating microglial expression of TLR7. **i** Neurons (NeuN) express TLR7 in later disease stages. **j** Astrocytes (GFAP) did not express TLR7. TLR7 upregulation is indicated in **k** cell quantifications, and **l** TLR3 is upregulated on transcriptional level, indicated by qPCR [*q* and *r*: controls (*n* = 7), stage 0 (*n* = 5), stage 1 (*n* = 6), and stage 2 (*n* = 5)]. Cell quantifications are represented as cells/mm^2^. qPCR data are indicated as − ΔCt for a better graphical representation. Statistical analyses are non-parametric, using the Kruskal–Wallis test with multiple comparisons to control group and Dunn’s post hoc correction, *p* values indicate results from multiple comparisons (**p < 0.01, ***p < 0.001). Data are represented as median with interquartile range. All scale bars correspond to 25 µm

The pronounced expression of inflammatory molecules as early as in PNs prompted us to investigate the upstream cascades. Inflammasome activation and IFN production are preceded by pathogen- or damage-associated molecular pattern (P- or DAMP) receptor signaling. Our microarray data revealed two significantly upregulated Toll-like receptors (TLRs), TLR3 and TLR7 (Fig. 5a). Whereas the tested anti-TLR3 antibodies did not show specific staining in our material, we could verify TLR7 immunohistochemically. TLR7 immunoreactivity in controls was weak (Fig. 5b), more abundant in PNs (Fig. 5c) and further increased in SNs (Fig. 5d). Besides a clear expression in microglia (Fig. 5e, h), with increased inflammation, TLR7 was also detected in neurons and seemed especially prominent in degenerating neurons (Fig. 5f, g, i). We did not find TLR7 expression in astrocytes (Fig. 5j). The absolute number of TLR7^+^ cells was not significantly higher in stage 0 than in controls (Fig. 5l). However, the TLR7 abundancy in microglia, as indicated by the densitometric measurement, was increased in stage 0 samples compared to controls (Fig. 5k). Moreover, the number of TLR7^+^ cells continuously increased from stages 0–2, at which point it was significantly upregulated (Fig. 5l). As our focus lies on microglia, we specifically quantified TLR7^+^ microglia, which showed a significant increase in stages 1 and 2 (Fig. 5l). mRNA levels of TLR3 were significantly upregulated in stage 2 (Fig. 5m).

### Gene expression profile of RE can be imitated by endosomal TLR stimulation in in vitro microglia

**Fig. 6:**
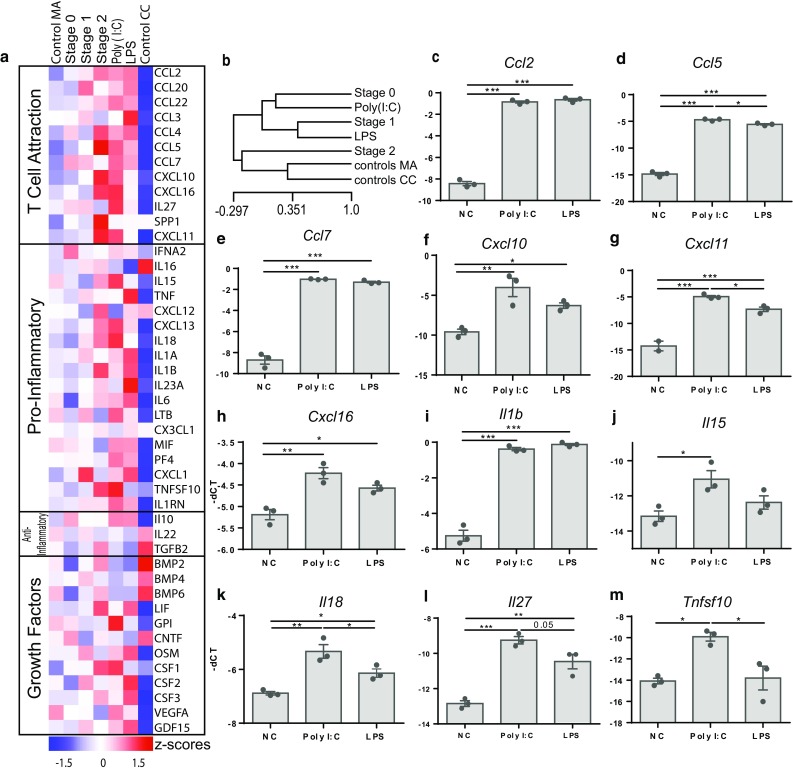
In vitro TLR3 stimulation reflects gene expression patterns observed in RE. **a** Differentially expressed genes of in vitro stimulated rat microglia cells compared to the expression values of RE. All values are represented in z scores. Control MA = control microarray (*n* = 7), stage 0 (*n* = 5), stage 1 (*n* = 6), stage 2 (*n* = 6), Poly(I:C) (*n* = 3), LPS (*n* = 3), and control CC = control cell culture (*n* = 3). **b** Hierarchical sample clustering of control MA, RE stage 0-2, Poly(I:C)- and LPS-treated microglia and control CC. Selected genes are depicted separately. **c***Ccl2* is significantly upregulated in both groups compared to controls, **d***Ccl5* is significantly elevated in Poly(I:C) group compared to LPS and control group, **e***Ccl7* is significantly upregulated in both groups compared to controls, **f** Cxcl10 was elevated in LPS and Poly(I:C) groups with a higher increase in the latter, **g***Cxcl11* was significantly elevated in Poly(I:C) compared to LPS and control groups, **h***Cxcl16* was elevated in LPS and Poly(I:C) with a higher increase in the latter, **i***Il1b* was increased in Poly(I:C) and LPS compared to controls, **j***Il15* was only increased in Poly(I:C) group, **k***Il18* was significantly elevated in Poly(I:C) compared to control and LPS groups, **l***Il27* was significantly elevated in Poly(I:C) compared to control, and **m***Tnfsf10* was significantly elevated in Poly(I:C) group compared to control and LPS groups (one-way ANOVA with multiple comparison and Tukey’s post hoc correction, *p* values represent results from multiple comparison, *p < 0.05, **p < 0.01, ***p < 0.001)

To further investigate the role of endosomal TLRs as initiating point for inflammatory reactions in RE, we performed in vitro stimulation of neonatal microglia cultures. We used the endosomal TLR3 agonist Poly(I:C) and the extracellular TLR4 agonist lipopolysaccharide (LPS) to identify differences between extracellular and endosomal TLR downstream pathways. Gene expression analysis by qPCR revealed that pro-inflammatory cytokines and chemokines were drastically upregulated in both Poly(I:C)- and LPS-treated groups (Fig. 6a). However, hierarchical sample clustering of RE stages 0–2 and the in vitro stimulated microglia pointed towards similar gene expression patterns between the early RE stages and TLR3-stimulated microglia (Fig. 6b). Especially pro-inflammatory mediators related to T-cell attraction, such as *Ccl5*, *Cxcl10*, *Cxcl11,* and *Cxcl16* (Fig. 6d, f–h), were expressed most strongly in Poly(I:C)-stimulated microglia and resembled the expression levels in RE (Fig. 6a). *Il15*, *Il18* and *Il27* (Fig. 6j–l), all of which are associated with the induction of an anti-viral phenotype of T cells [13], are prominently upregulated in Poly(I:C)-stimulated microglia. *Tnfsf10*, which activates NFκB signaling cascade and induces neuronal death [49], was drastically upregulated in TLR3-stimulated microglia (Fig. 6m). Other pro-inflammatory mediators, such as monocyte- and T-cell-attracting chemokines *Ccl2* (Fig. 6c) and *Ccl7* (Fig. 6e), and *Il1b* (Fig. 6i), were equally upregulated in the Poly(I:C) and LPS groups.

## Discussion

60 years after its first description [44], RE remains an enigmatic disease possibly resulting from autoimmune or anti-viral CTL-mediated mechanisms. Our transcriptome analysis of cortical RE tissues shows an early upregulation of multiple innate virus-related pathways at a stage (stage 1), when upregulation of CTL-associated pathways is still incomplete. These findings coincide with our immunohistochemical findings showing the presence of PN that lack CTLs, as early as stage 0. Comparison of the expression of D- PAMP receptors revealed only two significantly differentially expressed receptors, namely TLR3 and TLR7. Furthermore, immunohistochemical characterization of primary nodules revealed an upregulation of endosomal TLR7 in the earliest disease stage. In acute inflammation and degeneration, endosomal TLRs have been shown to sense endogenous ligands [12, 32, 48]. However, our data reveal the elevated levels of TLR7 in microglia in stage 0 cortici which do not contain CTLs or degenerating neurons. This is particularly interesting, since both TLR3 and TLR7 have been shown to be involved in recognition of viral RNA [24]. On the other hand, these TLRs were also shown to be involved in autoimmune diseases [23, 37]. An autoimmune etiology, however, seems not compatible to the unilateral affection in RE. TLR3 and TLR7 downstream signaling furthermore can lead to inflammasome gene activation [26, 34] shown already in stage 0 PNs. Furthermore, TLR3 and TLR7 can induce the high levels of interferon *α*/*β*, pro-inflammatory cytokines and chemokines [7, 11, 24], as shown in stage 1 and 2. In line with these results, in vitro microglia and human cortical transcriptome data show an upregulation of CTL-attracting chemokines upon TLR3 stimulation. The increased level of these chemokines may direct the CTLs to their neuronal targets, finally leading to the formation of SN in which CTLs and microglia are engaged in neuronal killing and epileptogenic synaptic stripping [5, 33]. IL-18, which is already increased in PNs, has been shown to play a role in virus clearance and to be a potent initiator of pro-inflammatory chemokine and cytokine expression [1, 38]. IL-18 can induce IFN-γ release from CTLs [1], which leads to the pSTAT1 expression pattern observed here and previously described in a virus-induced experimental model of RE [28, 36] and the increased IFN-γ levels reported in RE [41]. IFN-γ signaling by T-bet^+^ CTLs can then lead to the secretion of IL-1β, as previously shown for human monocytes [35], which was observed in this and previous studies [43]. In multiple sclerosis, microglial nodules (here called preactive lesions) can be found in the normal-appearing white matter [8, 18, 47]. Previously, we have shown that these nodules can express IL-1ß [9]. Besides the expression of small heat shock proteins [40] these preactive lesions are associated with the local presence of degenerating axons [47]. Another possibility, therefore, is that IL-1β induction may be similar in the preactive lesions of MS and the SNs in RE and may be the result of neurodegenerative mechanisms. This may also explain the loss of TMEM119 and especially P2RY12 in the SNs, since these homeostatic markers also have been shown downregulated in neurodegeneration [27]. The complex interactions between innate and adaptive immune system might explain why RE patients do not reach seizure freedom, as IL-1β and TLR3-activation has been shown to induce changes in neuronal excitability and reduce seizure threshold [53]. Summarized, our data strongly point towards TLR3 and TLR7 as initiator of inflammation in RE, possibly underlying a viral infection or a post-infectious autoimmune encephalitis [2]. We were able to delineate the primary pathological changes in RE by taking advantage of the surgical resection material of pathological differentiated lesions. We could therefore not only validate previous findings on the acute stage of RE (stage 2), such as increased IFN-γ, CCL5 or CXCL10 [28, 36, 41], but also describe the inflammatory processes leading up to them (Inflammatory interactions are summarized in Supplementary Fig. 1). As RE is considered a prime model disease for CTL-mediated encephalitis, these findings are not only important for RE but may also be relevant for the other T cell-mediated disease-forming microglial nodules such as viral or paraneoplastic encephalitides and MS.

## Electronic supplementary material

Below is the link to the electronic supplementary material.

Supplementary material 1 (DOCX 54 kb)

Supplementary material 2 (EPS 1147 kb)
