# Supplementary material for: Microglial nodules provide the environment for pathogenic T cells in human encephalitis
Source: Acta Neuropathol. 2019 Jan 20;137(4):619–35. doi: 10.1007/s00401-019-01958-5 (PMC6426829; doi:10.1007/s00401-019-01958-5)
Supplement: Supplementary file 1 — Supplementary material 1 (DOCX 54 kb) [file 401_2019_1958_MOESM1_ESM.docx]

**Supplementary table 1**: Patient Summary of all used samples

| Patient Nr | Stage  (Pardo et al) | Age  (years) | Disease Duration  (years) | Gender | Hemisphere  and anatomical region | Methods performed | DV200 | Diagnosis |  |
| --- | --- | --- | --- | --- | --- | --- | --- | --- | --- |
| 1 | 0 | 15.9 | 11.9 | f | L  T | MA, qPCR,  IHC | 82 | RE |  |
| 2 | 0 | 5.77 | 3.77 | m | R  T | MA, qPCR,  IHC | 85 | RE |  |
| 3 | 0 | 8.1 | 5.1 | f | L  T | MA, qPCR,  IHC | 83 | RE |  |
| 4 | 0 | *8.3* | *0.2* | f | L  T | MA, qPCR,  IHC | 85 | RE |  |
| 5 | 0 | 6.89 | 3.89 | m | R  T | MA, qPCR,  IHC | 74 | RE |  |
| 2 | 1 | 4.45 | 2.45 | m | R  T | MA, qPCR,  IHC | 78 | RE |  |
| 5 | 1 | 6.89 | 3.89 | m | R  T | MA, qPCR,  IHC | 69 | RE |  |
| 6 | 1 | 5.77 | 3.77 | m | R  T | MA, qPCR,  IHC | 72 | RE |  |
| 7 | 1 | 7.24 | 2.24 | f | L  T | MA, qPCR,  IHC | 80 | RE |  |
| 8 | 1 | 13.48 | 1.48 | f | L  T | MA, qPCR,  IHC | 89 | RE |  |
| 9 | 1 | 12.02 | 2.02 | f | R  T | MA, qPCR,  IHC | 74 | RE |  |
| 10 | 1 | 10.6 | 6 | m | R  T | qPCR, IHC |  | RE |  |
| 11 | 2 | 4.83 | 0.83 | m | R  F | MA, qPCR,  IHC | 86 | RE |  |
| 5 | 2 | 6.89 | 3.89 | m | R  T | MA, qPCR,  IHC | 76 | RE |  |
| 12 | 2 | 7.93 | 0.93 | f | L  T | MA, qPCR,  IHC | 90 | RE |  |
| 13 | 2 | 5.19 | 1.19 | f | R  T | MA, qPCR,  IHC | 72 | RE |  |
| 14 | 2 | 6.5 | 1 | m | R  T | MA, qPCR,  IHC | 78 | RE |  |
| 15 | 2 | *4.9* | 1 | m | R  T | MA, qPCR,  IHC | 86 | RE |  |
| 23 | 0 | 9.13 | 5.88 | m | R  ND | IHC |  | RE |  |
| 24 | 1 | 4.48 | 1.58 | m | R  FP | IHC |  | RE |  |
| 25 | 1 | 5.38 | 0.38 | f | L  FC | IHC |  | RE | |
| 26 | 0 | 29.3 | 17.3 | f | L  ND | IHC |  | RE | |
| 27 | 2 | 7.6 | 1 | f | L  T | IHC |  | RE | |
| 28 | 1 | 24.58 | 2.58 | f | R  F | IHC |  | RE | |
| 29 | 2 | 10.6 | 0.6 | f | L  T | IHC |  | RE | |
| 30 | 1 | 5.51 |  | m | L  FCP | IHC |  | RE | |
| Controls |  |  |  |  |  |  |  |  | |
| 16 | - | 4.85 | 0.85 | m | L  T | MA, qPCR,  IHC | 70 | DNT WHO I | |
| 17 | - | 7.71 | 4.21 | f | L  T | MA, qPCR,  IHC | 73 | Gangioglioma WHO I | |
| 18 | - | 9.18 | 8.68 | f | L  T | MA, qPCR,  IHC | 84 | Cavernom o.A. | |
| 19 | - | 7.25 | 5.25 | f | R  T | MA, qPCR,  IHC | 83 | Gangioglioma WHO I | |
| 20 | - | 9.14 | 2.14 | m | L  T | MA, qPCR,  IHC | 87 | DNT WHO I | |
| 21 | - | 8.97 | 8.22 | m | L  T | MA, qPCR,  IHC | 87 | Gangiogliom WHO I | |
| 22 | - | 4.44 | 4.04 | f | L  T | MA, qPCR,  IHC | 87 | Low Malignant Tumor | |

Abbreviations:m = male, f = female, L = left, R = right, T = temporal, F = frontal, FP = fronto-parietal, FC = fronto-central, FCP = fronto-centro-parietal, ND = not determined, MA = microarray, IHC = immunohistochemistry, RE = Rasmussen encephalitis, FCD = focal cortical dysplasia, DNT = dysembryoplastic neuroepithelial tumor

**Supplementary Table 2**: Differentially expressed pathways analyzed with gene-set enrichment analysis (GSEA) by the reactome software.

| Differentially Expressed Pathway (GSEA) | Stage 0 | | Stage 1 | | | Stage 2 | | |
| --- | --- | --- | --- | --- | --- | --- | --- | --- |
|  | Fold change | p-Value | Fold change | p-Value | FDR | Fold change | p-Value | FDR |
| Class I MHC mediated antigen processing and presentation | 1.56 | ns | 1.05 | ns | ns | 2.29 | 1.17E-05 | 8.52E-04 |
| Endosomal/Vacuolar pathway | NF |  | 1.57 | 1.18E-05 | 0.005 | 2.50 | 1.11E-16 | 1.62E-14 |
| Antigen processing-Cross presentation | 1.56 | ns | 1.57 | 0.01 | ns | 2.43 | 1.11E-16 | 1.62E-14 |
| Antigen Presentation: Folding, assembly and peptide loading of class I MHC | NF |  | 1.38 | 0.0131 | 0.01 | 2.52 | 1.11E-16 | 1.62E-14 |
| ER-Phagosome pathway | 1.56 | ns | 1.38 | 0.0053 | ns | 2.45 | 1.11E-16 | 1.62E-14 |
| Cytokine Signaling | 0.74 | ns | 1.49 | ns | ns | 2.44 | 1.24E-12 | 1.58E-10 |
| Interferon Signaling | 1.58 | ns | 1.57 | 0.0049 | ns | 2.67 | 1.11E-16 | 1.62E-14 |
| Interferon gamma signaling | 1.65 | ns | 1.52 | 4.33E-05 | 0.009 | 2.71 | 1.11E-16 | 1.62E-14 |
| Interferon alpha/beta signaling | 1.51 | ns | 1.66 | 1.02E-04 | 0.01 | 2.51 | 1.11E-16 | 1.62E-14 |
| Interleukin-1 processing | NF |  | NF |  |  | 1.60 | 0.04 | ns |
| Interleukin-6 signaling | NF |  | 2.15 | ns | ns | 2.90 | 0.023 | ns |
| Interleukin-10 signaling | NF |  | 1.95 | ns | ns | 2.41 | 0.005 | ns |
| Immunoregulatory interactions between a Lymphoid and a non-Lymphoid cell | NF |  | 1.59 | ns | ns | 2.31 | 1.56E-11 | 1.76E-09 |
| TCR Signaling | 1.59 | ns | 0.90 | ns | ns | 3.62 | 0.002 | ns |
| Downstream TCR signaling | 1.59 | ns | 0.79 | ns | ns | 3.78 | 0.002 | ns |
| Translocation of ZAP-70 to Immunological synapse | 1.65 | ns | 1.86 | Ns (0.06) | ns | 4.05 | 1.99E-09 | 2.03E-07 |
| Generation of second messenger molecules | 1.65 | ns | 1.83 | Ns (0.07) | ns | 3.84 | 4.64E-09 | 4.27E-07 |
| Phosphorylation of CD3 and TCR zeta chains | 1.65 | ns | 1.86 | Ns | ns | 4.05 | 7.30E-09 | 6.21E-07 |
| Costimulation by the CD28 family | 1.65 | ns | 1.86 | ns | ns | 3.74 | 5.64E-05 | 0.0038 |
| PD-1 signaling | 1.65 | ns | 1.86 | ns | ns | 4.05 | 1.10E-08 | 8.56E-07 |
| MHC class II antigen presentation | 1.65 | ns | 1.86 | ns | ns | 3.66 | 0.006 | ns |
| Complement Cascade | -0.975 | ns | 1.67 | ns | ns | 2.01 | ns | ns |
| Activation of C3 and C5 |  |  | 1.51 | ns | ns | 2.46 | 0.040 | ns |
| TGFβR2 MSI Framshift Mutation in Cancer |  |  |  |  |  | 1.75 | 0.023 | ns |

Abbreviations: NF = not found in GSEA analysis (pathway is not represented in our dataset), ns = not significant, FDR = false discovery rate

**Supplementary Table 3:** Functional Annotation clustering of genes enriched in Stage 1 with DAVID

| Annotation Cluster 1 | Enrichment Score: 3.54 | p-Value | Fold Change | FDR |
| --- | --- | --- | --- | --- |
| interferon-gamma-mediated signaling pathway |  | 8.0E-12 | 2.1E1 | 1.2E-8 |
| integral component of lumenal side of  endoplasmic reticulum membrane |  | 2.6E-9 | 3.4E1 | 3.3E-6 |
| ER to Golgi transport vesicle membrane |  | 8.3E-9 | 2.1E1 | 1.0E-5 |
| MHC class II protein complex |  | 1.7E-8 | 3.9E1 | 2.1E-5 |
| immune response |  | 3.4E-8 | 5.4E0 | 5.3E-5 |
| Herpes simplex infection |  | 4.9E-8 | 7.9E0 | 5.8E-5 |
| region of interest:Alpha-2 |  | 1.1E-7 | 4.8E1 | 1.7E-4 |
| region of interest:Alpha-1 |  | 1.1E-7 | 4.8E1 | 1.7E-4 |
| Immunoglobulin/major histocompatibility complex, conserved site |  | 5.3E-7 | 1.6E1 | 7.3E-4 |
| MHC class II, alpha/beta chain, N-terminal |  | 5.9E-7 | 3.5E1 | 8.2E-4 |
| IGc1 |  | 1.1E-6 | 1.4E1 | 1.2E-3 |
| Immunoglobulin C1-set |  | 1.3E-6 | 1.4E1 | 1.7E-3 |
| MHC classes I/II-like antigen recognition protein |  | 1.6E-6 | 1.9E1 | 2.2E-3 |
| Immunity |  | 2.1E-6 | 4.3E0 | 2.6E-3 |
| peptide antigen binding |  | 3.0E-6 | 2.6E1 | 3.9E-3 |
| antigen processing and presentation |  | 4.4E-6 | 1.6E1 | 6.9E-3 |
| domain:Ig-like C1-type |  | 4.7E-6 | 2.4E1 | 6.9E-3 |
| MHC class II receptor activity |  | 5.6E-6 | 4.0E1 | 7.4E-3 |
| region of interest:Connecting peptide |  | 6.4E-6 | 2.2E1 | 9.4E-3 |
| Staphylococcus aureus infection |  | 7.0E-6 | 1.4E1 | 8.3E-3 |
| Graft-versus-host disease |  | 9.1E-6 | 2.0E1 | 1.1E-2 |
| Allograft rejection |  | 1.6E-5 | 1.8E1 | 1.9E-2 |
| clathrin-coated endocytic vesicle membrane |  | 1.9E-5 | 1.8E1 | 2.4E-2 |
| Type I diabetes mellitus |  | 3.1E-5 | 1.6E1 | 3.6E-2 |

**Supplementary Table 4-7:** Functional Annotation clustering of genes enriched in Stage 2 with DAVID

| Annotation Cluster 1 | Enrichment Score: 15.09 | p-Value | Fold Change | FDR |
| --- | --- | --- | --- | --- |
| type I interferon signaling pathway |  | 9.4E-24 | 1.6E1 | 1.6E-20 |
| defense response to virus |  | 1.5E-19 | 7.9E0 | 2.5E-16 |
| Antiviral defense |  | 5.0E-15 | 8.6E0 | 6.8E-12 |
| negative regulation of viral genome replication |  | 1.7E-10 | 1.3E1 | 3.0E-7 |
| response to virus |  | 3.0E-10 | 6.8E0 | 5.2E-7 |

| Annotation Cluster 2 | Enrichment Score: 9.27 | p-Value | Fold Change | FDR |
| --- | --- | --- | --- | --- |
| interferon-gamma-mediated signaling pathway |  | 1.2E-35 | 1.9E1 | 2.1E-32 |
| Antigen processing and presentation |  | 1.8E-20 | 1.1E1 | 2.2E-17 |
| domain:Ig-like C1-type |  | 1.1E-19 | 2.3E1 | 1.8E-16 |
| Graft-versus-host disease |  | 5.6E-19 | 1.8E1 | 7.2E-16 |
| integral component of lumenal side of  endoplasmic reticulum membrane |  | 1.1E-18 | 2.3E1 | 1.6E-15 |
| Staphylococcus aureus infection |  | 4.4E-18 | 1.2E1 | 5.6E-15 |
| MHC class II protein complex |  | 1.0E-17 | 2.7E1 | 1.4E-14 |
| antigen processing and presentation |  | 3.6E-17 | 1.4E1 | 6.2E-14 |
| Type I diabetes mellitus |  | 1.5E-16 | 1.4E1 | 1.4E-13 |
| Immunoglobulin/major histocompatibility complex, conserved site |  | 2.2E-16 | 1.3E1 | 3.4E-13 |
| MHC class II, alpha/beta chain, N-terminal |  | 2.4E-16 | 2.6E1 | 3.4E-13 |
| Allograft rejection |  | 2.5E-16 | 1.5E1 | 2.8E-13 |
| MHC classes I/II-like antigen recognition protein |  | 2.8E-16 | 1.6E1 | 5.1E-13 |
| Viral myocarditis |  | 3.0E-16 | 1.1E1 | 4.2E-13 |
| peptide antigen binding |  | 8.6E-16 | 2.1E1 | 1.3E-12 |
| Herpes simplex infection |  | 2.2E-15 | 5.5E0 | 2.8E-12 |
| Immunoglobulin C1-set |  | 3.2E-15 | 1.2E1 | 5.0E-12 |
| ER to Golgi transport vesicle membrane |  | 5.5E-15 | 1.4E1 | 7.4E-12 |
| Cell adhesion molecules (CAMs) |  | 5.8E-15 | 6.2E0 | 7.3E-12 |
| IGc1 |  | 7.3E-15 | 1.1E1 | 9.0E-12 |
| MHC II |  | 1.5E-14 | 2.9E1 | 2.1E-11 |
| region of interest:Connecting peptide |  | 1.9E-14 | 1.8E1 | 3.1E-11 |
| antigen processing and presentation of peptide  antigen via MHC class II |  | 2.6E-14 | 2.8E1 | 4.5E-11 |
| Influenza A |  | 1.8E-13 | 5.3E0 | 2.3E-10 |
| Phagosome |  | 1.9E-13 | 5.7E0 | 2.4E-10 |
| Autoimmune thyroid disease |  | 2.3E-13 | 1.1E1 | 2.9E-10 |
| MHC class II receptor activity |  | 2.3E-13 | 2.9E1 | 3.4E-10 |
| Rheumatoid arthritis |  | 3.3E-12 | 7.3E0 | 4.3E-9 |
| MHC class II, beta chain, N-terminal |  | 1.7E-11 | 2.6E1 | 2.6E-8 |
| region of interest:Alpha-1 |  | 2.7E-11 | 2.6E1 | 4.5E-8 |
| region of interest:Alpha-2 |  | 2.7E-11 | 2.6E1 | 4.5E-8 |
| SM00921 |  | 4.2E-11 | 2.4E1 | 5.3E-8 |
| MHC I |  | 5.0E-10 | 3.3E1 | 6.8E-7 |
| Leishmaniasis |  | 6.1E-10 | 7.3E0 | 7.8E-7 |
| Asthma |  | 1.1E-9 | 1.2E1 | 1.4E-6 |
| Inflammatory bowel disease (IBD) |  | 1.2E-9 | 7.6E0 | 1.5E-6 |
| Intestinal immune network for IgA production |  | 1.7E-9 | 9.1E0 | 2.2E-6 |
| MHC class I protein complex |  | 2.0E-9 | 2.9E1 | 2.7E-6 |
| Tuberculosis |  | 2.1E-9 | 4.3E0 | 2.6E-6 |
| clathrin-coated endocytic vesicle membrane |  | 4.0E-9 | 1.1E1 | 5.5E-6 |
| MHC class I, alpha chain, alpha1/alpha2 |  | 4.4E-9 | 2.6E1 | 6.8E-6 |
| region of interest:Beta-1 |  | 5.4E-9 | 3.6E1 | 8.9E-6 |
| region of interest:Beta-2 |  | 5.4E-9 | 3.6E1 | 8.9E-6 |
| Systemic lupus erythematosus |  | 9.3E-9 | 4.8E0 | 1.2E-5 |
| antigen processing and presentation of  exogenous peptide antigen via MHC class II |  | 9.7E-9 | 6.9E0 | 1.7E-5 |
| antigen processing and presentation of  exogenous peptide antigen via MHC class I, TAP-independent |  | 2.0E-8 | 3.1E1 | 3.5E-5 |
| transport vesicle membrane |  | 2.5E-8 | 1.1E1 | 3.5E-5 |
| antigen processing and presentation of peptide  antigen via MHC class I |  | 3.5E-8 | 1.3E1 | 6.0E-5 |
| antigen processing and presentation of exogenous  peptide antigen via MHC class I, TAP-dependent |  | 4.9E-8 | 8.1E0 | 8.5E-5 |
| Epstein-Barr virus infection |  | 6.4E-8 | 4.8E0 | 8.1E-5 |
| endocytic vesicle membrane |  | 8.9E-8 | 7.7E0 | 1.2E-4 |
| Lysosome |  | 1.1E-7 | 3.8E0 | 1.5E-4 |
| MHC class I, alpha chain, C-terminal |  | 2.0E-7 | 3.4E1 | 3.1E-4 |
| phagocytic vesicle membrane |  | 2.4E-7 | 8.0E0 | 3.2E-4 |
| T cell costimulation |  | 5.7E-7 | 6.6E0 | 9.8E-4 |
| T cell receptor signaling pathway |  | 1.1E-6 | 4.5E0 | 1.9E-3 |
| region of interest:Alpha-3 |  | 1.9E-6 | 2.5E1 | 3.1E-3 |
| peptide antigen assembly with MHC class II  protein complex |  | 2.0E-6 | 3.9E1 | 3.4E-3 |
| Toxoplasmosis |  | 2.3E-6 | 4.4E0 | 2.9E-3 |
| MHC class I-like antigen recognition |  | 4.6E-6 | 1.1E1 | 7.2E-3 |
| HTLV-I infection |  | 7.4E-6 | 2.9E0 | 9.4E-3 |
| trans-Golgi network membrane |  | 7.9E-6 | 5.7E0 | 1.1E-2 |
| early endosome membrane |  | 7.9E-6 | 4.7E0 | 1.1E-2 |
| Immunoglobulin-like domain |  | 1.4E-5 | 2.1E0 | 2.1E-2 |
| Golgi membrane |  | 2.2E-5 | 2.3E0 | 3.0E-2 |

| Annotation Cluster 3 | Enrichment Score: 7 | p-Value | Fold Change | FDR |
| --- | --- | --- | --- | --- |
| signal peptide |  | 1.5E-13 | 1.8E0 | 2.5E-10 |
| disulfide bond |  | 5.9E-13 | 1.9E0 | 9.7E-10 |
| Disulfide bond |  | 2.5E-12 | 1.8E0 | 3.3E-9 |
| Glycoprotein |  | 9.8E-11 | 1.6E0 | 1.3E-7 |
| Signal |  | 3.6E-10 | 1.6E0 | 4.9E-7 |
| glycosylation site:N-linked (GlcNAc...) |  | 5.2E-9 | 1.5E0 | 8.6E-6 |
| topological domain:Cytoplasmic |  | 2.7E-8 | 1.6E0 | 4.5E-5 |
| plasma membrane |  | 2.2E-7 | 1.5E0 | 2.9E-4 |
| topological domain:Extracellular |  | 3.8E-7 | 1.6E0 | 6.3E-4 |
| integral component of plasma membrane |  | 2.2E-6 | 1.8E0 | 3.0E-3 |
| transmembrane region |  | 1.6E-5 | 1.3E0 | 2.7E-2 |
| Cell membrane |  | 2.1E-5 | 1.5E0 | 2.8E-2 |
| Receptor |  | 2.8E-5 | 1.7E0 | 3.8E-2 |

| Annotation Cluster 4 | Enrichment Score: 2.61 | p-Value | Fold Change | FDR |
| --- | --- | --- | --- | --- |
| Chemotaxis |  | 8.1E-8 | 6.4E0 | 1.1E-4 |
| chemotaxis |  | 4.6E-7 | 5.2E0 | 7.9E-4 |
| cell chemotaxis |  | 5.0E-6 | 6.7E0 | 8.6E-3 |
| Cytokine-cytokine receptor interaction |  | 3.8E-5 | 2.8E0 | 4.8E-2 |

**Supplementary Table 8: Primer specifications**

| Gene | Forward Primer | Reverse Primer |
| --- | --- | --- |
| GAPDH | 5´ ATATTGTTGCCATCAATGACCC 3´ | 5´ ATGACAAGCTTCCCGTTCTC 3´ |
| GAPDH | 5´ CATTTCCTGGTATGACAACGA 3´ | 5´ CTTCCTCTTGTGCTCTTGCT 3´ |
| SDHA | 5’-GGCAGGGTTTAATACAGCAT-3’ | 5’-TAGAAATGCCACCTCCAGTT-3’ |
| IL1ß | 5’ CATTGCTCAAGTGTCTGAAGC 3’ | 5’ GGAGCACTTCATCTGTTTAGG 3’ |
| IL18 | 5’ CCTTTAAGGAAATGAATCCTCCTG 3’ | 5’ CATCTTATTATCATGTCCTGGGAC 3’ |
| Caspase1 | 5’ TGATGCTATTAAGAAAGCCCAC 3’ | 5’ GAAACATTATCTGGTGTGGAAGAG 3’ |
| TLR3 | 5' TCATCCAACAGAATCATGAGAC 3’ | 5' CTTCATGGCTAACAGTGCAC 3’ |
| TLR7 | PrimePCR SYBR Green Assay  qHsaCED0048113 |  |

**Supplementary Table 9: Protocol details for immunohistochemical stainings**

| Target | Host  Species | | Pretreatment | | | Dilution | | Company/Ref.Nr. | | | |
| --- | --- | --- | --- | --- | --- | --- | --- | --- | --- | --- | --- |
| NeuN | Ms | | C | | | 1:500 | | Chemicon, MAB377 | | | |
| CD68 | Ms | | E8.5 | | | 1:50 | | Dako Agilent, M0814 | | | |
| c-FOS | Rb | | E8.5 | | | 1:1500 + CSA enhancement | | Abcam, #ab208942 | | | |
| Iba1 | Rb | | E8.5 | | | 1:3000 (1:1500 for fluo) | | Wako, #019-19741 | | | |
| CD3 | Rb | | E8.5 | | | 1:500 | | Dako, A0452 | | | |
| IL-1β | Goat | | | E8.5 | | 1:250 | | | Santa Cruz, #sc1250 | | |
| Caspase-1 | Rb | | | E8.5 | | 1:5000 | | | Proteintech, #22915-1-A | | |
| IL-18 | Rb | | | E8.5 | | 1:2000 | | | Abcam, #ab191152 | | |
| TLR7 | Rb | | | E8.5 | | 1:100 (1:300+CSA for fluo) | | | Abcam, #ab124928 | | |
| pSTAT1 | Rb | | | E8.5 | | 1:2000 + CSA enhancement | | | Cell Signalling, #9167 | | |
| t-bet | Ms | | C | | | 1:150 + CSA enhancement | | | | Abcam, #91109 | |
| GFAP | Ms | | C | | | 1:100 | | | | Neomarkers 8/15 | |
| P2RY12 | Rb | | E8.5 | | | 1:100 | | | | Gift from Dr. Oleg Butovsky, Harvard Medical School | |
| TMEM119 | Rb | | E8.5 | | | 1:500 | | | | Sigma, HPA051870 | |
| HLA-DR | Ms | | C | | | 1:100 | | | | Dako, clone CR3/43 | |
| Bi-α-Ms | | Donkey | | |  | | 1:1500 | | | | Jackson Immuno Research, #705-065-150 |
| Bi-α-Rb | | Donkey | | |  | | 1:2000 | | | | Jackon Immuno Research, #711-165-152 |
| Bi-α-goat | | Donkey | | |  | | 1:500 | | | | Jackson Immuno Research, #705-065-147 |
| Cy3-α-Ms | | Donkey | | |  | | 1:100 | | | | Jackson Immuno Research, #715-165-151 |
| Cy5-α-Rb | | Donkey | | |  | | 1:200 | | | | Jackson Immuno Research, #711-175-152 |
| Streptavidin-Cy2 | |  | | |  | | 1:100 | | | | Jackson ImmunoResearch  #016-220-084 |

MS = mouse, Rb = rabbit, Bi = biotinylated, α = anti, CSA = tyramide signal enhancement
